# *Trypanosoma cruzi* (Agent of Chagas Disease) in Sympatric Human and Dog Populations in “Colonias” of the Lower Rio Grande Valley of Texas

**DOI:** 10.4269/ajtmh.16-0789

**Published:** 2017-04-05

**Authors:** Rachel Curtis-Robles, Italo B. Zecca, Valery Roman-Cruz, Ester S. Carbajal, Lisa D. Auckland, Isidore Flores, Ann V. Millard, Sarah A. Hamer

**Affiliations:** 1 College of Veterinary Medicine and Biomedical Sciences, Texas A&M University, College Station, Texas; 2 Department of Entomology, Texas A&M University, College Station, Texas; 3 International Valley Health Institute, Edinburg, Texas; 4 School of Public Health, Texas A&M Health Science Center, McAllen, Texas

## Abstract

The zoonotic, vector-borne parasite *Trypanosoma cruzi* causes Chagas disease throughout the Americas, but human and veterinary health burdens in the United States are unknown. We conducted a cross-sectional prevalence study in indigent, medically underserved human and cohabiting canine populations of seven south Texas border communities, known as colonias. Defining positivity as those samples that were positive on two or more independent tests, we found 1.3% seroprevalence in 233 humans, including one child born in the United States with only short-duration travel to Mexico. Additionally, a single child with no travel outside south Texas was positive on only a single test. Among 209 dogs, seroprevalence was 19.6%, but adjusted to 31.6% when including those dogs positive on only one test and extrapolating potential false negatives. Parasite DNA was detected in five dogs, indicating potential parasitemia. Seropositive dogs lived in all sampled colonias with no difference in odds of positivity across age, sex, or breed. Colonia residents collected two adult *Triatoma gerstaeckeri* and one nymph triatomine from around their homes; one of three bugs was infected with *T. cruzi*, and blood meal hosts were molecularly determined to include dog, human, and raccoon. Dogs and the infected vector all harbored *T. cruzi* discrete typing unit I, which has previously been implicated in human disease in the United States. Colonias harbor active *T. cruzi* transmission cycles and should be a priority in outreach and vector control initiatives.

## Introduction

The vector-borne parasite *Trypanosoma cruzi* causes Chagas disease in humans and dogs throughout the Americas. An estimated 6 million people are infected throughout Latin America^1^; and estimates of infected immigrants living in the United States range from approximately 72,000 to over 300,000.^2^,^3^ Locally acquired human cases have been recognized in the southern United States, where Texas has documented transmission.^4^–^6^ Vector-borne transmission is via infected triatomine “kissing bug” insects found throughout the Americas, including the southern United States.^7^,^8^ The parasite may also be transmitted congenitally, via blood transfusion or organ transplant, and through ingestion of infected bugs or contaminated foods.^8^ Infected persons and dogs may develop acute nonspecific disease, followed by a prolonged asymptomatic period; chronic disease is characterized by parasite damage to tissues, including the heart.^1^ In some cases, mortality has been documented in canines during the acute phase.^9^,^10^ No human or canine vaccine exists, and antiparasitic treatment options are limited.

*Trypanosoma cruzi* infection in canines is well described in the southern United States,^10^–^14^ though the degree to which canine infection reflects human disease risk in the United States is unknown. Studies throughout the Americas have shown dogs to be important reservoir hosts for, and potential sentinels of, *T. cruzi*.^15^ Dogs in a household have been shown to be a risk factor for vector presence in houses,^16^–^18^ and their presence is associated with increased odds of infected bugs and people within households in South American studies.^17^,^19^,^20^ However, canine infection has been shown to vary widely across studies, likely influenced by local transmission dynamics (sylvatic versus domestic or peridomestic), circulating *T. cruzi* strain types, recent vector control initiatives, and diagnostic methods and reporting.^15^ Canines have been proposed as sentinels for *T. cruzi* infection in humans,^11^,^21^,^22^ although the degree to which canine infection is indicative of human risk is dependent upon consideration of local transmission dynamics.^15^

Along the United States–Mexico border, approximately 1.7 million individuals inhabit “colonias”—economically distressed and unincorporated border communities with inadequate sewer, water, and/or electric services.^23^ Although the term “colonia” most directly translates to “community” or “neighborhood” in Spanish throughout many parts of Latin America, in the United States the term is used most specifically to describe impoverished rural settlements along the United States–Mexico border region (see 23–25 for more detailed information regarding colonias). In Texas, there are an estimated 1,800–2,300 designated colonias inhabited by 400,000–500,000 predominantly Hispanic individuals.^24^ The actual number of colonias and their population size may be greater due to unrecorded colonias and undocumented or transient individuals. Such Hispanic and Latino populations in the United States may be at higher risk for Chagas disease if they have emigrated from countries with high disease burdens, given that the vast majority of individuals in the United States reported to have Chagas disease were born in or have travel histories to endemic regions in Latin America.^2^ Health disparities related to limited health education, poor access to health care, low incomes, and poor housing structure may be important risk factors for locally acquired infections. We suggest that people living in colonias may be at high risk for vector-borne infections because substandard housing surrounded by wild habitats may be conducive to vector colonization of homes. Further, because many dogs in colonias typically live outdoors and roam freely, these dogs may be at increased risk of exposure to kissing bugs and infected wildlife that may serve as sources of infective stages of *T. cruzi*.^26^–^29^ Given these risk factors, the objective of this study was to determine *T. cruzi* infection prevalence in sympatric human and dog populations of rural colonias in south Texas.

## Materials and Methods

**Figure 1.**
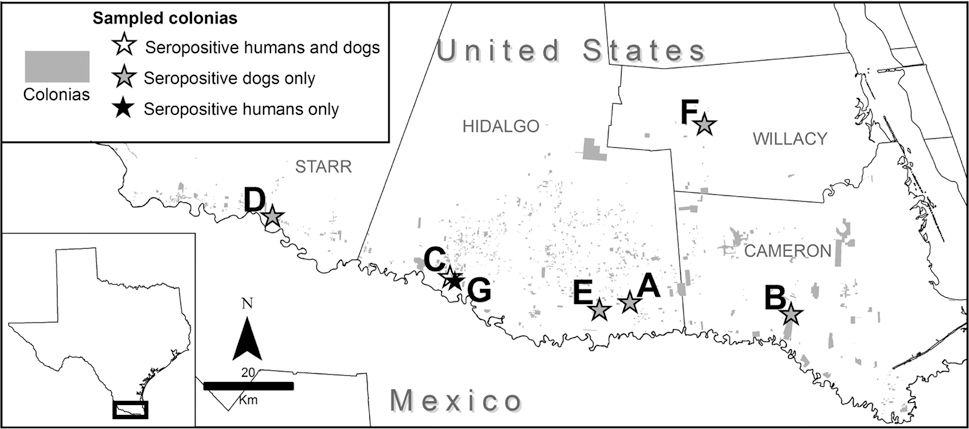
Colonias in south Texas. Seven colonias in the four south Texas counties of the Rio Grande Valley were sampled; seropositive individuals are defined as those that were positive on at least two tests. One additional human (in Colonia B) was seropositive on only one test.

Humans and dogs in south Texas colonias (Figure 1

) were sampled from July to November 2015, coincident with seasonal triatomine activity across Texas.^7^ Colonias were selected to represent the lower Rio Grande Valley (Hidalgo, Cameron, Starr, and Willacy counties) with selection based, in some cases, on preexisting relationships with the project promotora (community health worker) and, in one case (Colonia B), geographic location near previous report of Chagas disease in dogs.^12^ All of the colonias sampled were located in border counties or in a county adjacent to a border county. The total number of households per sampled colonia ranged from 30 to 600 and approximately 5–25% of the households were sampled (5% from largest colonia and 25% from smallest colonia). The majority of the sampled colonias were surrounded by agricultural fields, undeveloped land, or natural areas. Most of the sampled colonias had unpaved or damaged paved roads. Housing within each colonia varied from severely dilapidated mobile homes to new brick homes. The most commonly observed housing consisted of mobile homes with poorly constructed add-ons or partially completed homes in which the construction appeared to have ceased. Electricity, running water, and sewage systems were observed in the majority of the sampled colonia households, although some homes operated under unreliable services due to unregulated electricity lines, water pipes, and sewage systems.

Study documents including consent forms and outreach materials were available to participants in English and Spanish. To facilitate study enrollment, the promotora alerted each community days prior to sampling and provided outreach materials about kissing bugs and Chagas disease to 10–20 households in each colonia. Colonia members who received information were encouraged to invite other members of their colonia. Sampling of humans (age ≥ 4 years) and dogs (age ≥ 3 months) occurred at a central location in each colonia; human-only sampling occurred in one colonia (Colonia G) that prohibited canines. Children under the age of 4 years were not sampled because our team did not perform pediatric evaluations that would have been required prior to blood draws. After informed consent was obtained from adult participants and from parents or legal guardians of minors, 2–5 mL of blood was obtained from participating individuals and their canines. Rabies vaccination of dogs after blood draws was provided as an incentive for owners to bring their dogs for sampling. A brief survey of each human participant included sociodemographic questions and details of travel history. Participants were shown pictures of kissing bugs and resin-encapsulated kissing bug specimens and asked to indicate if and where they had previously seen kissing bug vectors. Kissing bugs found by participants during the study were accepted for identification and *T. cruzi* testing. Human and canine sampling and survey protocols were approved by Texas A&M University Institutional Review Board and Institutional Animal Care and Use Committee, respectively.

Human and canine serum or plasma samples were initially tested for anti-*T. cruzi* antibodies using Chagas Stat-Pak (Chembio Diagnostic Systems, Inc., Medford, NY). This rapid immunochromatographic test has shown high sensitivity (> 95%) and specificity (> 95%) in human samples, compared with other serological techniques^30^–^35^; however, others have found considerable variation in and lower sensitivity (26.6–87.5%) depending upon geographic region.^36^ Chagas Stat-Pak has been used previously for research in dogs^11^,^13^,^37^; one study calculated sensitivity and specificity of 100% (however, high values may have been the result of not counting faint bands as positive).^13^ All positive samples, and a subset of negative dog samples, were then tested with Chagas Detect Plus Rapid Test (InBios International, Inc., Seattle, WA), a rapid immunochromatographic test based on specific recombinant antigens designed for use in humans^38^ (showing 96–99% sensitivity and 97–99% specificity^39^). Both the Chagas Stat-Pak and InBios Chagas Detect Plus Rapid tests have integrated test controls; in all cases, “control” bands appeared as expected. When sample volumes allowed, human samples that initially tested positive were tested for IgG antibodies at Mayo Medical Laboratories (Rochester, MN) using Hemagen Chagas ELISA kit (Hemagen Diagnostics, Inc., Columbia, MD), and canine samples that initially tested positive were tested for IgG antibodies using indirect fluorescent antibody (IFA) testing (Texas Veterinary Medical Diagnostic Laboratory, College Station, TX). As per Texas Veterinary Medical Diagnostic Laboratory reporting standard, samples positive at dilutions of 1:20 or greater were considered IFA-positive. The IFA test is currently the most widely available test for diagnosis of canine anti-*T. cruzi* antibodies in the southern United States. Serological tests were often performed after freezing and thawing of serum with periods of storage that exceeded 1 month. Considering potential variability in detection sensitivity and specificity, we defined as positive only those individuals that were positive on two or more independent tests. We do, however, also present the data for those individuals that reacted on only a single test because the true infection status of individuals with discordant test results is currently unknown.

To directly detect *T. cruzi*, DNA extracted from the buffy coat (E.Z.N.A. kit; Omega Bio-Tek, Norcross, GA) was subjected to quantitative polymerase chain reaction (PCR) to amplify nuclear *T. cruzi* DNA^40^,^41^ using a Stratagene MxPro3000 instrument (Agilent Technologies, Santa Clara, CA) and previously described thermocycling parameters,^40^ except with a 3-minute initial denaturation. Reactions contained 5 μL of DNA, primers at a final concentration of 0.75 μM each, 0.25 μM of probe, and iTaq Universal Probes Supermix (BioRad Laboratories, Hercules, CA), in a total volume of 20 μL. Successful amplification was assured by visually checking machine-calculated thresholds and reaction curves. A Ct value less than 31 was positive, based on internal laboratory validations using serial dilutions of *T. cruzi* to calculate limit of detection and PCR efficiency for this assay. We used a multiplex probe-based assay amplifying the spliced leader intergenic region (SL-IR) to determine *T. cruzi* discrete typing units (DTUs).^42^ Assays were run with a 20-μL reaction volume using the Multiplex PCR Kit (Qiagen, Valencia, CA) on a BioRad CFX96 following published protocol.^42^ DNA-negative water controls and positive controls of DNA extracted from Sylvio X10 CL4 (ATCC 50800, American Type Culture Collection, Manassas, VA) and *T. cruzi*-infected *Triatoma sanguisuga* from Texas were included in all PCR batches.

Kissing bug specimens were identified morphologically to species,^43^ sexed, and dissected. DNA from bug hindguts was extracted and subjected to quantitative PCR^40^ to determine infection status; parasite DTU was ascertained as described above.^42^ To determine blood meal source, bug hindgut DNA was subjected to PCR amplifications targeting cytochrome *b* DNA.^44^–^47^ We used an iterative process in which extracted DNA was subjected to multiple separate PCRs for the same or different regions of cytochrome *b* for three reasons. First, given degraded DNA from these insects that were collected and stored by colonia residents, some attempts to amplify were negative even when residual blood traces were observed in the hindgut, and the overall chance of assigning a blood meal host was greater with multiple attempts. Second, rerunning all samples was especially important when sequences comprised human DNA. Although we decontaminated the external surface of insects prior to dissection, we are aware that contamination may occur and human DNA could serve as a likely source; accordingly, rerunning PCR and resequencing across multiple genetic regions afforded an independent opportunity to confirm the presence of human DNA. Finally, the iterative process afforded the opportunity to generate different amplicons and sequences representing different host species from the same insects that could be interpreted as mixed blood meals from feeding on multiple host species. PCR using the “herp” primer set^44^,^45^ included 3 μL template DNA, primers at final concentrations of 0.66 μM each, and FailSafe PCR Enzyme Mix with PreMix E (Epicentre, Madison, WI) in a final reaction volume of 50 μL using previously described cycling conditions.^44^ PCR using the “BM” primer set^45^,^47^,^48^ included 1.5 μL template DNA, primers at final concentrations of 0.66 μM each, and FailSafe PCR Enzyme Mix with PreMix E (Epicentre) in a final reaction volume of 15 μL using previously described cycling conditions.^47^ PCR using the “mammalian a” primer set^45^–^47^ included 1.5 μL template DNA, primers at final concentrations of 0.66 μM each, and FailSafe PCR Enzyme Mix with PreMix E (Epicentre) in a final reaction volume of 15 μL using previously described cycling conditions.^46^,^47^ DNA-negative water controls and positive controls of DNA extracted from white-tailed deer (*Odocoileus virginianus*), opossum (*Didelphis virginiana*), or cynomolgus macaque (*Macaca fascicularis*) were included in all PCR batches. Amplicons were visualized on 1.5% agarose gels with ethidium bromide, and amplicons of expected product sizes were sequenced using Sanger sequencing (Eton Bioscience Inc., San Diego, CA) in both directions. Forward and reverse strands were aligned and the consensus region was compared with published sequences using Basic Local Alignment Search Tool (National Center for Biotechnology Information, U.S. National Library of Medicine).

To evaluate the relationship between putative risk factors and canine seroprevalence, bivariable analysis using χ^2^ or Fisher's exact test was used. Variables were colonia (A–F), canine age (< 2 years, ≥ 2 years), sex, and breed group (per American Kennel Club: herding, sporting, terrier, toy, and “other”; “other” was comprised of breed groups with relatively small sample sizes including hound, mix, nonsporting, and working breeds). Logistic regression models were built using Program R^49^ using the lme4 package to further investigate risk factors with *P* < 0.25 in the initial screening or those with justification for inclusion based on published data. Odds ratios and 95% confidence intervals were calculated. Descriptive statistics for human data were calculated, but the low number of seropositive humans precluded further analysis.

## Results

We collected samples from 233 humans and 209 dogs from 143 households, of which 77 households (53.8%) had both humans and dogs contribute samples to the study. Of human participants, 146 were female (62.7%), and 87 were male (37.3%). There were 81 children (4–17 years of age) sampled (34.8% of total). Mean age of females was 35.4 years (standard deviation [SD]: 19.7 years; range: 4–83 years), and of males was 27.0 years (SD: 22.1 years; range: 4–80 years). Most participants surveyed (78.0%) spoke only Spanish or “more Spanish than English” at home. A total of 44 (18.9%) individuals confirmed having seen kissing bugs. Bug sightings were mainly stated to be in Texas (84.1%) with the remainder seen in Mexico (15.9%). Of Texas sightings, 62.2% occurred inside the respondent's home or outdoors in the colonia. The remaining sightings occurred in other towns, friends' homes, ranches, and/or workplace environments.

Anti-*T. cruzi* antibodies were present in three of 233 (1.3%) human blood samples as evidenced by reaction in ≥ 2 independent tests, and one additional human was positive on a single test only (Tables 1 and 2). Two positive individuals—a 68-year-old man (GH03) and a 40-year-old woman (GH16)—were from the same colonia (Colonia G). The other positive individual was a 13-year-old girl from Colonia C (CH29; Table 2). A 13-year-old boy from Colonia B (BH54) was associated with a single positive test result. Epidemiological follow-up revealed the two positive adults were born in Mexico, and the positive child was born in Texas to a mother that was determined to be seronegative in our study. All three positive individuals reported travel to Mexico ranging from < 1-week biannual trips to 8-week trips every 5 years. The child associated with a single positive test result (BH54) reported no travel outside south Texas and was born to a mother that was determined to be seronegative in our study (Table 2). Additionally, the 13-year old child of GH16 was determined to be seronegative in our study. Blood samples from 186 humans had sufficient volume for DNA extraction and attempted PCR amplification; none had evidence of *T. cruzi* DNA.

Of 209 dogs, 96 were female (45.9%) and 113 were male (54.1%). The majority (128 dogs, 61.2%) were small-breed dogs, weighing less than 9 kg. Dogs had a mean age of 2.8 years (SD: 2.53 years; range: 3 months to 13 years). A diversity of breed groups and mixes were represented, with the largest representation comprised of toy breeds (83 dogs, 39.7% of total). In particular, Chihuahua and Chihuahua-mix dogs represented 34.0% of the total.

Anti-*T. cruzi* antibodies were present in 41 of 209 (19.6%) canine blood samples based on positivity on ≥ 2 tests. Seropositive dogs were found in all six colonias with dogs (Table 3). Seven seropositive dogs were in single-dog households; 16 seropositive dogs were the only seropositive dog in a household from which multiple dogs were tested, and 18 seropositive dogs were from eight multidog households having two or three seropositive dogs. One seropositive dog lived in the same house as the human (BH54) that was positive on only one serologic assay. In bivariable analyses, only the relationship between colonia and seropositivity was associated with a *P* value below the cutoff (within-colonia seroprevalence ranged from 9.4% to 31.8%, *P* = 0.130, Fisher's exact test), but all four putative risk factors were retained in the regression model based on previous findings.^10^,^50^,^51^ A logistic regression model did not reveal differences in the odds of canine seropositivity by sex, age, or breed group (Table 3). Among the colonias, the odds of seropositivity were significantly lower among dogs residing in the Colonia F relative to dogs in the referent (OR = 0.191, 95% CI = 0.050–0.688, *P* = 0.012; Table 3).

Of 157 dogs that tested negative on the Chagas Stat-Pak, 11 were randomly selected and subjected to additional testing, of which a single dog (9.1%) was positive on IFA (at a 1:20 dilution) but negative on Chagas Detect Plus Rapid test. Applying this prevalence of potentially positive samples to the population of Chagas Stat-Pak-negative dogs, we extrapolate 14 additional dogs might have been seropositive. When summing the 41 dogs positive on at least 2 tests, the 11 dogs positive on only a single test, and this estimate of potential false negatives, a more liberal estimate of canine seroprevalence is 66 of 209 dogs (31.6%).

Of 184 canines with sufficient blood sample for DNA extraction and PCR amplification, five dogs (2.7%) had evidence of *T. cruzi* DNA. All five also reacted on serological assays, with four positive on Chagas Stat-Pak plus at least one other assay, and one dog positive on Chagas Stat-Pak but negative on IFA and Chagas Detect Plus Rapid assays. Three PCR-positive dogs were from Colonia A (*N* = 20), including two from the same household, and the remaining two were from Colonia B (*N* = 54) and D (*N* = 27). No PCR-positive dogs were detected in colonias C (*N* = 13), E (*N* = 23), or F (*N* = 47). Four of the five dogs were sampled in July and one was sampled in November. The SL-IR PCR allowed for ascertaining the parasite DTU in four of these dogs; all were infected with TcI.

Three kissing bugs were collected from around homes in colonias during the study. COL1 was an adult female *Triatoma gerstaeckeri*. The submitter of COL1 indicated that the insect was feeding on his arm and that he regularly found kissing bugs around his raised wooden home. COL1 was positive for *T. cruzi* DTU TcI. When sequencing the amplicons from the “BM” primer set, a consensus sequence of 220 base pairs (bp) most closely matched (100% identity) human sequences. On the second independent attempt using the same primer set, a consensus sequence of 232 bp most closely matched (99% identity) raccoon (*Procyon lotor*). Amplification of this bug sample was attempted twice using the “herp” primer set and once using the “mammalian a” primer set, but these attempts were unsuccessful. COL2 was an adult female *T. gerstaeckeri* found by a colonia resident who reported regularly seeing and crushing triatomines both inside and outside of her home. COL2 was negative for *T. cruzi*. When sequencing amplicons from the “BM” primer set, a consensus sequence of 218 bp was identical to human sequences (100% identity). On the second independent attempt using the same primer set, a consensus sequence of 245 bp was again identical to human (100% identity). Additional attempts, twice using the “herp” primer set and once using the “mammalian a” primer set, were unsuccessful. Finally, COL3 was a nymph found feeding on a dog. COL3 was negative for *T. cruzi*. When sequencing amplicons from the “herp” primer set, a consensus sequence of 82 bp was identical to *Canis lupus* (100% identity). Although the bidirectionally confirmed consensus sequence was only 82 bp, each of the individual forward and reverse sequences were longer (> 220 bp), and each individually also matched *C. lupus* with 100% identity. Additional attempts, once using the “herp” primer set, once using the “mammalian a” primer set, and twice using the “BM” primer set, were unsuccessful.

## Discussion

Humans and dogs from seven colonias in south Texas were tested for anti-*T. cruzi* antibodies and *T. cruzi* DNA. We detected 1.3% seroprevalence in 233 humans, and 19.6% seroprevalence in 209 cohabiting canines of diverse breeds, although adjustment for potential false negatives suggests canine seroprevalence may approach 31.6%. Given that approximately 500,000 humans live in Texas colonias,^24^ extrapolation of our study findings suggest 6,500–8,500 infected people may reside in Texas colonias. No human samples had evidence of *T. cruzi* DNA, whereas five dogs (2.7% of 184 tested) also had evidence of *T. cruzi* DNA in their blood, likely indicating potential parasitemia. Further, adult and nymph triatomine vectors, including an infected insect and insects with evidence of having fed on humans, dog, and raccoon, were collected in the colonias. Cumulatively, these results highlight active transmission cycles of *T. cruzi* in south Texas colonias.

In contrast to the many studies testing populations throughout Latin America for *T. cruzi* infection, studies in the United States are limited, and Texas is one of few U.S. states with history of such studies. A study in the 1960s found 1.4% seroprevalence in 500 children from south central Texas.^52^ A 1970s study of people along the south Texas border area showed 0.8% seroprevalence.^29^ More recently, a study from 2008 to 2012 found a seroprevalence of 0.015% in blood donors.^53^ In the colonias, the seroprevalence we detected (1.3%) is two orders of magnitude higher than the recent finding in Texas blood donors, likely reflective of sociodemographic and health status differences between colonia populations and the general blood donor population.

Although autochthonous infection is possible in all cases of humans associated with positive test results, it may be more likely that infections were acquired in Mexico for at least three of these four individuals, where there is an estimated infection prevalence of 0.78%.^1^ The two seropositive adults were born in Mexico and moved to the United States in adulthood, with sporadic, short-duration visits to Mexico. The seropositive child was born in Texas and had visited Mexico for short durations (< 1 week) approximately twice per year. Although *T. cruzi* transmission could have occurred during such visits, vector-borne transmission of *T. cruzi* is inefficient—one mathematical model indicates 900–4,000 contacts with infected bugs are necessary for vector-borne parasite transmission.^54^ On the other hand, there is at least one documented case of infection in a child in a house with very low vector presence.^55^ The child (BH54) with a positive result on only a single test was born in Texas, with no travel history outside the Rio Grande Valley, and his mother was seronegative, suggesting a potential autochthonous infection, although further testing would be needed to confirm infection.

**Figure 2.**
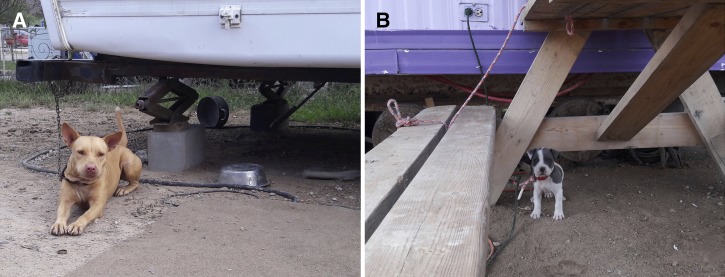
(A) Adult and (B) puppy pitbull mix dogs under homes in colonias, Rio Grande Valley of Texas, 2015. Many dogs in colonias are kept outside, where they rest and sleep under the raised houses.

Although sampling was demographically biased, with mainly mothers and children sampled since men were usually working away from the home, women in Latino communities traditionally play a major role in health-care decision-making and accessing health services^14^ and therefore were a priority in our outreach efforts. A study of Latino immigrants in the state of Georgia described minimal Chagas awareness, education, screening, and treatment in participants due to cultural and language barriers, economic limitations, fear of deportation, and cultural preference for traditional medicines.^56^ Colonia residents are similarly likely to face such factors inhibiting Chagas disease awareness, in addition to additional decidedly unique characteristics of colonias (see 24, 57) that may further limit Chagas disease awareness, including poverty, poor infrastructure, and lack of access to health care and education. Others have noted that colonia residents, known to be primarily Spanish-speaking individuals, are more likely to have poorer physical health than the general U.S. population.^58^ A prior serologic study of Chagas disease in the general Texan population in the 1960s specifically noted that houses with wood frame construction raised off the ground were conducive to close proximity of infected wildlife^52^; construction of many houses in colonias we sampled met this description, with dogs commonly observed to rest or sleep under the houses (Figure 2

).

Our finding of 19.6% seroprevalence in canines is higher than previous studies in south Texas, which showed 6.7–8.8% seroprevalence in stray or shelter dogs.^11^,^12^,^29^ Canine infection has been shown to vary widely across studies throughout the Americas, likely influenced by local transmission dynamics (sylvatic versus domestic or peridomestic), circulating *T. cruzi* strain types, recent vector control initiatives, and diagnostic methods and reporting.^15^ Dog presence in households has been shown to be a risk factor for vector presence,^16^–^18^ and is associated with increased odds of infected bugs and people within households in South American studies.^17^,^19^,^20^,^59^ Canines have been proposed as sentinels for *T. cruzi* infection in humans^11^,^15^,^21^,^22^; however, the degree to which canine infection is indicative of human risk is dependent upon consideration of local transmission dynamics.^15^ Despite evidence of widespread canine exposure,^10^ the degree to which infected dogs reflect a risk of human disease in the very different ecological settings across south Texas and the United States is largely unknown. The risk of vector-borne transmission of *T. cruzi* in humans can increase when animals live in or around households,^16^–^19^ which is the case for most colonia residents. However, the three seropositive humans in our study resided in a colonia that did not allow dog ownership or did not report owning a dog; in contrast, a seropositive canine resided on the property of the child associated with a single positive test result (BH54), indicating the surrounding environment supported potential autochthonous infection.

We found 2.7% of canines had *T. cruzi* DNA in their blood. Although molecular methods alone do not conclusively confirm parasitemia, these observations may indicate infectious levels of circulating parasites.^60^ Only DTU TcI was found in PCR-positive dogs, which contrasts limited prior findings of mainly TcIV in U.S. dogs.^61^ The few U.S. human infections for which parasite DTU has been determined have revealed exclusively TcI.^61^

Due to financial limitations and cultural factors, dog ownership in colonias is typically regarded differently than in the general U.S. population. Dog owners who consider their dogs as “family” are more likely to provide regular care to their animals, and in comparison to the general population, low-income Hispanics—who compose the majority of colonia populations—are less likely to consider their dogs as “family.”^62^ Canines in colonias are often kept tied or loose outdoors, resulting in stray dog packs that may cause public health hazards.^63^ Weak infrastructure in colonias further limits county government efforts of stray animal control.^23^ The outdoor-stray lifestyle of colonia dogs is likely to increase their risk of exposure to vectors and sylvatic reservoirs, thus increasing *T. cruzi* transmission risk. Though travel histories of dogs were not known, based on the subset of seropositive dogs with owners who completed a survey (*N* = 31), the ages of nearly all (93.5%) the dogs were less than the period of time their owners had lived in the colonias or immediate area, suggesting locally acquired infections.

The lack of age, sex, and breed predispositions for canine Chagas seropositivity in our data suggests that all dogs that share environments with infected bugs are at risk. When compared with dogs in Colonia A (the model referent), dogs in Colonia F had significantly lower odds of being seropositive. The county of Colonia F has a lower percentage of Hispanics and is more rural than the counties of the other sampled colonias,^64^ congruent with a prior study found lower seroprevalence in rural dogs as compared with urban dogs.^65^

Incongruences across different serological tests are a challenge in Chagas disease diagnostics,^32^,^66^ and findings in this study are no exception. Differences in test design and interpretation, local circulating *T. cruzi* strains, antigens used, and sample integrity (which may be compromised in repeated thaw/freeze cycles) have been suggested as potentially affecting classification of samples as positive versus negative.^36^,^67^ An important consideration is that the low seroprevalence in sampled humans limits the positive predictive value of the diagnostic tests. To reduce the number of potential false positives in this study, we used the criteria of positive results on at least two independent tests to indicate a positive individual. Additionally, although population-level epidemiological research may proceed with presentation of ranges of estimated seroprevalence based on different diagnostics, individual-level diagnosis and prognosis suffer from lack of clear understanding of what positivity on only one test signifies. Individuals in this study who were positive on at least one serological test were given contact information of local medical providers able to assist with further testing and treatment options.

Triatomines submitted by participants during the study included a *T. cruzi-*infected *T. gerstaeckeri* and evidence of the triatomines having fed on human, dog, and raccoon; these findings underscore active transmission risk among humans, dogs, and wildlife within colonias. We found success in assigning blood meal hosts using an iterative process in which each insect DNA extract was subjected to multiple PCRs; this approach afforded confidence through being able to assess reproducibility of results. In addition to evidence of feeding on a dog in one bug and a human in another bug, DNA from a third insect generated both human and raccoon sequences. Given this insect fed a minimum of six times (at least once per nymphal instar plus adult stage), but likely many more times, evidence of mixed hosts in the hindgut is biologically reasonable and in this case underscores transmission risk between wildlife and humans.

Using a One Health framework, our observations of infected dogs, humans, and triatomines within shared households highlight the border colonias as at-risk communities for Chagas disease. Education and outreach campaigns with associated vector control measures should appropriately focus on these communities with high priority.

## Figures and Tables

**Table 1:** Human *Trypanosoma cruzi* antibody testing results (*N* = 233) in the colonias of south Texas, 2015

Colonia	County	Month sampled	No. tested	No. seropositive	Seroprevalence (%)
A	Hidalgo	July	11	0	0
B	Cameron	July	54	0*	0
C	Hidalgo	July	28	1	3.6
D	Starr	November	35	0	0
E	Hidalgo	July	45	0	0
F	Willacy	November	44	0	0
G	Hidalgo	July	16	2	12.5
Total			233	3	1.3

Seroprevalence estimates are based on positivity based on two or more independent test results.

* One individual was positive on a single test only and therefore did not meet the positivity criterion for the study.

**Table 2:** Details from surveys of four individuals associated with at least one *Trypanosoma cruzi* positive serological test result, Texas, 2015

Serologic test results (Chagas Stat-Pak/Chagas Detect/Hemagen)	GH03	GH16	CH29	BH54
	+/+/+	+/+/−	+/+/not run	+/−/−
Colonia, county	G, Hidalgo	G, Hidalgo	C, Hidalgo	B, Cameron
Sex	Male	Female	Female	Male
Ethnicity	Hispanic	Hispanic	Hispanic	Hispanic
Age, years	68	40	13	13
Occupation	Agriculture and oil industry	Homemaker	Student	Student
Dogs included in study	No	No	No	1 seropositive dog and 1 seronegative dog
Kissing bug sighting in Texas	No	No	Yes	No
Birthplace	Mexico	Mexico	United States	United States
Time lived in United States	20 years	5 years	13 years	13 years
Time lived in present colonia	1 year	1 year	13 years	1 year
Most recent prior living location	Rio Grande Valley	Mission, TX	NA	Brownsville, TX
Travel within Texas or the United States	Yes (Louisiana—10 years ago)	No	No	No
Travel to other countries	Yes (Mexico)	Yes (Mexico)	Yes (Mexico)	No
Duration of travel	8 weeks	1–2 weeks	< 1 week	NA
Frequency of travel to Mexico	Every 5 years	Every 5 years	Twice per year	NA
Seasonality of travel to Mexico	Summer	Summer	Summer	NA
Maternal birthplace	Mexico	Mexico	Mexico	Mexico
Serostatus of mother	Unknown	Unknown	Seronegative	Seronegative
History of blood transfusion or organ transplant	No	No	No	No
Medical history involving heart or gastrointestinal disease	Hypertension	No	No	No

NA = not applicable.

**Table 3:** Logistic regression model output to identify signalment predictors of *Trypanosoma cruzi* seropositivity among 209 dogs in the colonias of south Texas, 2015

Demographic factor	No. tested	No. seropositive	Seroprevalence (%)	Odds ratio	95% confidence interval	*P* value
Colonia						
A	22	7	31.8		Referent	
B	54	12	22.2	0.592	0.180–2.007	0.390
C	14	4	28.6	0.945	0.192–4.285	0.942
D	32	7	21.9	0.510	0.135–1.864	0.304
E	23	5	21.7	0.547	0.122–2.279	0.413
F	64	6	9.4	0.191	0.050–0.688	**0.012**
Age						
< 2 years	93	18	19.4		Referent	
≥ 2 years	116	23	19.8	1.038	0.511–2.110	0.917
Sex						
Female	96	19	19.8		Referent	
Male	113	22	19.5	1.029	0.508–2.086	0.936
Breed group						
Herding	30	7	23.3		Referent	
Sporting	19	3	15.8	0.661	0.145–3.017	0.593
Terrier	39	6	15.4	0.601	0.177–2.044	0.415
Toy	83	17	20.7	0.884	0.319–2.452	0.813
Other	38	8	21.1	0.918	0.284–2.970	0.887
Total	209	41	19.6*			

* This estimate increases to 31.6% when including extrapolated potential false negatives.
